# Postoperative complications of pediatric dental general anesthesia procedure provided in Jeddah hospitals, Saudi Arabia

**DOI:** 10.1186/1472-6831-9-6

**Published:** 2009-02-19

**Authors:** Najat Farsi, Rania Ba'akdah, Abdulaziz Boker, Abdullah Almushayt

**Affiliations:** 1 Department of Preventive Dental Sciences, Pediatric Dentistry Division, Faculty of Dentistry, Jeddah, Saudi Arabia; 2 National Guard Hospital, Jeddah, Saudi Arabia; 3 Department of Anesthesia and Critical Care, King Abdulaziz University Hospital, Jeddah, Saudi Arabia

## Abstract

**Background:**

Review of post-operative morbidity reports for pediatric dental care under general anesthesia (GA) show great variations. Until now, no morbidity data has been available to estimate the safety of pediatric patients under GA for dental rehabilitation in Saudi Arabia. The purposes of this study were to (1) investigate post-operative complications associated with dental care under GA and (2) correlate morbidity reports with patient's characteristics, dental procedures, and hospital protocol.

**Methods:**

Study sample included 90 children attending GA for dental treatment at major governmental hospitals in Jeddah. Data were collected from every patient on three occasions, intra-operatively at the operating room, and post-operatively via phone calls in the first and third days after operation.

**Results:**

Results showed that 99% of the children had one or more complaints in the first day in contrast to only 33% in the third day. Inability to eat (86%), sleepiness (71%), and pain (48%) were the most common complaints in the first day, followed by bleeding (40%), drowsiness (39%), sore throat (34%), vomiting (26%), psychological changes (24%), fever (21%), cough (12%), and nausea (8%). A great significant complaints reduction was reported by the third post-operative day. Age, gender, admission type of the patients and GA duration were the factors that showed a significant relationship with post-operative complaints.

**Conclusion:**

Post-operative morbidity was common, but mostly of mild severity and limited to the first day. Hospital staff efforts should be directed to control commonly reported postoperative complaints.

## Background

Children's anxiety plays an important role in their dental treatment. Pediatric dentists provide comprehensive dental treatment for their patients in a conscious state. However, some of the very young individuals, or those suffering extreme anxiety, medical impairment, and mental or physical disabilities could only be treated under GA. In a survey about the used behavior techniques by dentists in Saudi Arabia, sixty percent of pediatric dentists in Saudi Arabia reported using GA to treat their patients [1].

Safety of the children who need dental treatment under GA remains the major limiting factor for GA use. United Kingdom General Dental Council guidelines stated that "GA is a procedure which is never without a risk" [2]. Children's safety during and after the pediatric dental GA procedure can be estimated by the complication rate reported. Reports of post-operative morbidities with pediatric dental rehabilitation under GA were found in the range of negligible to more than 90% of patients [3-5]. Post-operative pain was the most common and long lasting complaint reported [3,6,7].

Sleep alteration in the first night after GA was commonly reported [3,7]. Although, sleepiness was not considered as a major problem, it usually raised the concern of the parents about their child's post-operative safety. Among postoperative complaints, sleepiness was most commonly reported 1 hour after GA, however, the number of children complaining of sleepiness tailed off very quickly after discharge.

In an early report by Holt and others [3] nausea was reported in 21% of the patients and vomiting in 20%. More recently, post-operative nausea and vomiting was rarely reported [5,8].

Psychological changes after GA procedure was reported in different forms; crying, trouble sleeping, nightmares, and changes in children's ability to eat [3,6]. Post-operative epistaxis, sore throat, and hoarseness were not uncommon in children [3,9]. Post-operative dental bleeding was reported high during recovery (71%) especially for extraction cases; however, it was greatly decreased after discharged [5,8].

From previous reports, the morbidity figures were used to determine the patient risk and played a prominent role to identify its etiological factors, accurately set malpractice rates, establish regulations, and determine means of prevention. Many factors were reported to trigger development of post-operative complications such as patient age, patient medical status, patient dental needs, staff experience, pre-medication used, total anesthetic time, intubation difficulty, and anesthetic medications [3-8,10-13]

In Saudi Arabia, pediatric dentists have no local reports about mortality or morbidity of pediatric dental rehabilitation under GA. They depend on international studies to provide information on patient safety and risk. A majority of those studies were reported by maxillofacial surgeons. The difference in patient age and the type of treatments between pediatric dental rehabilitation procedures and maxillofacial surgeon procedures under GA may significantly alter the difference in mortality and morbidity rate. Additionally, the problems of previously reported post-operative complications for pediatric patients under GA were the retrospective designs used to study post-operative complications in most of the researches.

The lack of local data about pediatric dental GA procedure post-operative morbidity was the main purpose of this investigation. The purposes of this study were to establish specific morbidity data for pediatric dental GA procedure at three hospitals representing three different governmental sectors in Jeddah, Saudi Arabia, and determine the relationship of child demographics, medical condition, hospital protocols, and dental procedures with morbidity.

## Methods

The study was designed to be a prospective observational study supported by a pre-formulated questionnaire for post-operative complaints of children. The study was approved by the ethical committee at the Preventive Dental Sciences Department at Faculty of Dentistry, King Abdulaziz University in Jeddah.

The study included three governmental hospitals in Jeddah offering free dental rehabilitation under GA for pediatric patients in the Ministry of Health Hospital, the University Hospital, and the National Guard Hospital. All children attending for dental rehabilitation under GA were considered eligible to participate in the study. During the period of the study, 24 weeks, ninety six children were approached. Six children were excluded for the following reasons: two children with American Society of Anesthesiologist physical status (ASA-PS) classification more than II, one child for combined surgery, one child with parents who had no home or mobile phone to be contacted post-operatively, one participant who answered only one post-operative call and one who refused to participate in the study. Ninety children who attended for dental rehabilitation under GA (30 from each hospital) were included in the study after obtaining informed written parental consent.

Before commencing the main study, a pilot study was performed using 10 children, not included in the main study. Data from pilot study were used to refine the questionnaire. Data were collected by a single investigator. Preoperative data were recorded from the patient records including; Patient age, gender, medical condition, and admission type (in-patient, out-patient). Dental data were reported as mean of treated teeth and the different types of treatment provided. Post-operative complications were assessed through telephone calls to the patient's mother/guardian by the investigator using a pre-formed questionnaire, after the first day (after 24 hours) and after the third day (after 72 hours).

The onsets of reporting the post-operative complaints in previous studies varied from the recovery area, during journey home, first day, third day, and up to one month after dental GA. After three days of GA, it was reported that complaints were limited only to 10% of the patients [7]. This guided our study to observe the possible post-operative complications up to three days only. In the present study parents assessed their children's complaints (not the children themselves) because of the younger and medically compromised children who might not be able to communicate. The wide use and good responses in the pilot study with telephone interview supported its use in our study. The questionnaire format was developed based on the reported previous complaints and common response to pilot study. Also, parents were given the opportunity to add new comments or complaints noticed.

The questionnaire covered the following items: did the child have pain, bleeding from dental origin, sore throat, fever, vomiting, inability to eat, sleepiness, drowsiness, cough, nausea or psychological changes? The severity of the first 5 complaints was evaluated using a verbal descriptive severity scale. The scores were none/mild/moderate/severe [14]. The instant the child regained physical activity was also reported.

Information was analyzed using Statistical Package for Social Sciences Program SPSS (10.01) software. The study included descriptive and analytical data. A P-value of less than 0.05 was considered statistically significant.

## Results

The demographic characteristics of the sample are presented in Table 1. The children's age range was from 1 to 13 years with the mean 5.3 ± 2.1 years. Most of the treated children were healthy (71%) and only 21 children (23%) were medically compromised. Neurological/mental problems were the most common medical reasons for patients treatment under GA (11 patients) followed by asthma (4), hematological problem (2), Down syndrome (2) and cardiac problem (2). The mean anesthesia time for GA procedures was 124.6 min (range = 10 – 295 min). Sevoflurane inhalation anesthetic agent was used for almost all of the patient (96%), while isoflurane was used for 4% of the patients. Post-operatively, oral analgesic (15 mg/kg oral paracetamol) was prescribed to all of the patients. Only 4 patients, because of severe pain at home, received rescue analgesic in the form of 20 mg/kg paracetamol rectally.

**Table 1:** Frequency distribution of demographic variables of the sample.

	Gender		Age (years)		Medical condition		Anesthesia duration (min)		Admission type	
	
Variables	Male	Female	≤ 5	> 5	Healthy	Medically compromised	< 125	≥ 125	In patient	Out patient
	
N(%)	46 (51.5)	44 (48.4)	56 (62.2)	34 (37.8)	69 (76.7)	21 (23.3)	47 (52.2)	43 (47.8)	46 (51.1)	44 (48.9)

All patients were treated by pediatric dentist consultants or pediatric dentist residents under direct supervision of pediatric dentist consultants. The mean number of present teeth was 20 ± 2.2 teeth, while the mean number of treated teeth was 14 ± 3.8. The most frequent treatment was; extraction (5 ± 4.6) followed by colored fillings (4.2 ± 3.2), stainless steel crowns (SSCs) (3.8 ± 3), and pulpotomies (2.6 ± 2.7). The least applied dental treatments were sealants (0.5 ± 0.7), amalgam fillings (0.2 ± 0.1) and pulpectomies (0.2 ± 0.3). Sutures were used in 9 (10%) of the patients.

Table 2 presents the frequency of pediatric complications at first and third post-operative days of GA procedures. Presence of one or more post-operative complaints at day one was very high (98.9%). The most common complaints reported were: inability to eat normally (85.5%), sleepiness (71.1%), and dental pain (47.8%) followed by dental bleeding (40%), drowsiness (38.9%), sore throat (34.4%), vomiting (25.6%), psychological changes (24.4%), and fever (21.1%). The least reported complaints at day one were nausea (7.8%) and cough (12.2%). By the third day, 33.3% of the children had one or more complaints. Half of the complained patients had dental pain (16.7%). In-ability to eat normally was present in 12.2%, and a sore throat 7.8%. Analysis of the differences between first and third day complaints showed that by day three, all patients' complaints were significantly reduced.

**Table 2:** The frequency of morbidity with pediatric dentistry GA procedure at first and third day post-operatively.

	**COMPLAINTS AT DAY 1**	**COMPLAINTS AT DAY 3**	P-VALUE*
		
**POST-OPERATIVE COMPLAINTS**	**N = 90** **n (%)**	**N = 90** **n (%)**	
Inability to eat	77 (85.5)	11 (12.2)	< 0.001
Sleepiness	64 (71.1)	0	< 0.001
Dental pain	43(47.8)	15 (16.7)	< 0.001
Dental bleeding	36 (40.0)	5 (5.6)	< 0.001
Drowsiness	35 (38.9)	0	< 0.001
Sore throat	31 (34.4)	7 (7.8)	< 0.001
Vomiting	23 (25.6)	1 (1.1)	< 0.001
Psychological changes	22 (24.4)	3 (3.3)	< 0.001
Fever	19 (21.1)	2 (2.2)	< 0.001
Cough	11 (12.2)	4 (4.4)	0.031
Nausea	7 (7.8)	0	0.016

P value of the difference between day 1 and 3 Using McNemar test

After discharge, only three patients went back to the hospitals for medical consultation of postoperative cough. However, none of them required hospital admission. The nature of psychological changes was reported mostly in the form of poor sleep or dreams. Other children showed excessive crying, involuntary urination and unspecified fear.

**Figure 1:**
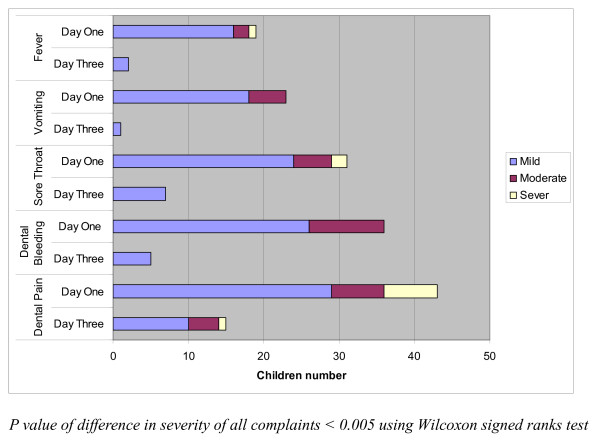
**Frequency distribution of complaints severity for pediatric dentistry GA participants at first and third day post-operatively**.

Figure 1 presents the frequency of complaint severity scores at day one and day three after GA procedure. At day one, most of the reported complaints were of mild or moderate severity. A severe score reported at day one was for dental pain, sore throat, and fever complaints only. The majority of day three complaints severity was limited to the mild category and none of the complaints was reported as severe except for one patient with dental pain. The statistical analysis for the severity difference from day one to day three showed a significant reduction in complaints severity.

Table 3 presents the relationship between major post-operative morbidity at day one and patient age, gender, medical condition, hospital admission type and anesthesia duration. Age and gender of the patients were found to be significantly related to some post-operative complications. Fever, drowsiness, and dental pain were significantly presented among younger children; however, dental bleeding was reported more significantly among older children. Postoperative pain occurred more frequently in males than females. Medically compromised patients did not significantly differ in postoperative complaints from healthy patients.

**Table 3:** Relationship between day one post-operative complaints and age, gender, medical condition, admission type and anesthetic duration.

Post-operative complaints	Age (years)		Gender		Medical condition		Admission type		Anesthesia duration (min)	
	
	≤ 5 n(%)	> 5 n(%)	M n(%)	F n(%)	H n(%)	MC n(%)	In n(%)	Out n(%)	< 125 n(%)	≥ 125 n(%)
Inability to eat (77)	50 (64.9)	27 (35.1)	37 (48.1)	40 (51.9)	59 (76.6)	18 (24.4)	36 (46.8)	41 * (53.2)	39 (50.6)	38 (49.4)
Sleepiness (64)	41 (64.1)	23 (35.9)	34 (53.1)	30 (46.9)	52 (81.3)	12 (18.8)	41 (64.1)	23 (35.9)	29 (45.3)	35 * (54.7)
Dental pain (43)	33 (76.7)	10 ** (23.3)	27 (62.8)	16 * (37.2)	34 (79.1)	9 (20.9)	19 (44.2)	24 * (55.8)	20 (46.5)	23 (53.5)
Dental bleeding (36)	17 (47.2)	19 * (52.8)	15 (41.7)	21 (58.3)	24 (66.7)	12 (33.3)	23 (63.9)	13 * (36.1)	24 (66.7)	12 * (33.3)
Drowsiness (35)	26 (74.3)	9 * (25.7)	21 (60)	14 (40)	28 (80)	7 (20)	14 (40.0)	21 (60.0)	13 (37.1)	22 * (62.9)
Sore throat (31)	20 (64.5)	11 (35.5)	13 (41.9)	18 (58.1)	27 (87.1)	4 (12.9)	16 (51.6)	15 (48.4)	16 (51.6)	15 (48.4)
Vomiting (23)	16 (69.6)	7 (30.4)	11 (47.8)	12 (52.2)	19 (82.6)	4 (17.4)	9 (39.1)	14 (60.9)	15 (65.2)	8 (34.8)
Psychological changes (22)	16 (54.5)	6 (27.3)	9 (40.9)	13 (59.1)	17 (77.3)	5 (22.7)	14 (63.6)	8 (36.4)	6 (27.3)	16 * (72.7)
Fever (19)	16 (84.2)	3 * (15.8)	10 (52.6)	9 (47.4)	16 (84.2)	3 (15.8)	10 (52.6)	9 (47.4)	9 (47.4)	10 (52.6)

(M = male, F = female, H = healthy, MC = medically compromise. In = in-patient, Out = out-patient. Using Ch-square test P value of difference between groups *P < 0.05, **P < 0.01

Regarding the relationship between participant's post-operative complaints and admission types, there was a significant difference between "in" and "out-patients" in their inability to eat and bleeding complaints. Out-patient admission participants reported significantly more inability to eat. However, in-patient reported significantly higher dental bleeding.

Longer anesthesia duration was significantly related to more sleepiness, drowsiness and psychological changes but to less bleeding complaints.

'The large number of statistical comparisons in table 3 might have led to a risk of Type – 1 error and the variables that were significant only at a level between 0.05 and 0.01 must be regarded with caution

**Figure 2:**
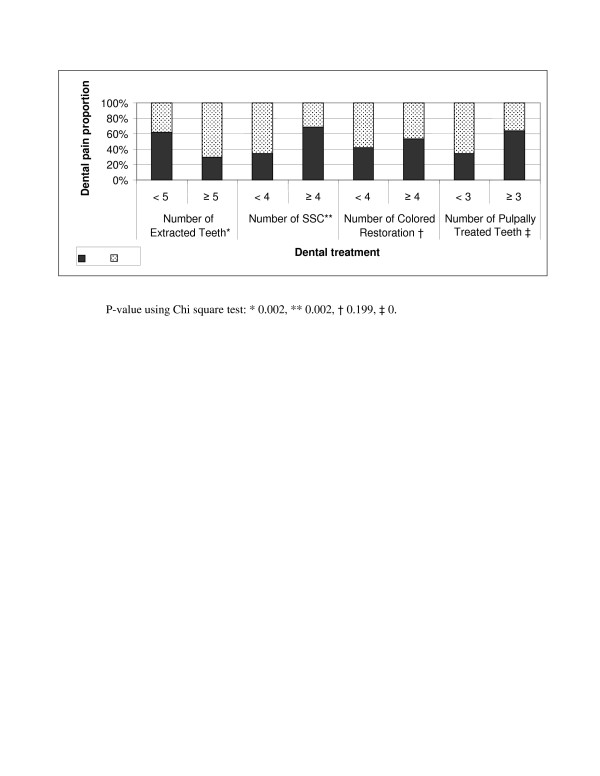
**Relationship of dental treatment and postoperative dental pain**.

The relationship between dental procedure type and development of postoperative dental pain is shown in figure 2. The tooth colored filling was the only procedure of non-significant value in relation to development of postoperative pain. Complaint of bleeding was significantly related to number of extracted teeth (P < 0.005).

**Figure 3:**
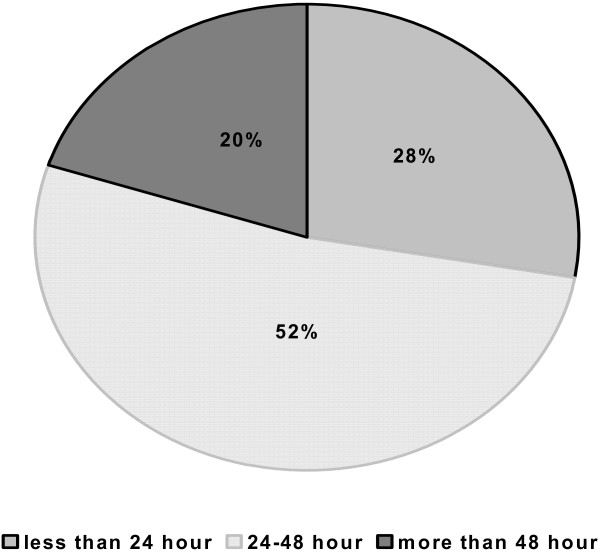
**Frequency distribution of participants regain of physical activity**.

Only 28% of the patients regained their physical activity within the first day. Most of the children (52%) regained their physical activity within the 2^nd ^day and 20% after the third day post-operatively (figure 3).

## Discussion

This study mainly aimed to investigate the nature, frequency, severity, and duration of post-operative complaints following pediatric dental GA procedure. In general, during the first three days post-operatively, children postoperative complaints did not require hospitalization.

Nearly all patients reported complaints in the first day post-operatively similar to some reports [3,6], but greater than others [4,15]. The difference between the present and previous studies may be due to the longer duration, 24 hours, allowed to report complaints during the first day after discharge, which gave a greater opportunity for the symptoms to occur and be reported. However, some studies reported complaints at one or separate occasions such as within the recovery room, on the journey home, after reaching home, and the first night, which divided the first day complaints into a shorter period and consequently lesser complaints frequency.

By the third day, post-operative complaints were reported by one third of the patients and mainly of mild severity. This was higher than third day reports in previous studies [4,7]. This might be due to the larger number of complaints stated in the questionnaire to be observed by the parents in addition to any other complaints noticed

The frequency of dental pain reported in this study was higher than the study of Vinckier and others [5], which reported no children pain in the recovery period by usage of analgesic suppositories. On the other hand, the severity of pain reported in this study was limited to the mild-moderate category at day one and it was significantly reduced by the third day similar to previous reports [7]. Additionally, our study found that most of the children regained their physical activity by the second postoperative day. These findings confirmed the benefit of good analgesic control with pediatric dental GA procedure, specially, during the first day similar to previous studies [4,13,16].

In this study, dental pain was found more among younger male patients. This may be due to the slow rate of boy's psychological maturation that makes them unable to tolerate pain at a younger age than females.

Although high post-operative bleeding (71%) was previously reported with extraction under GA [6], our results reported that only 40% of the children had bleeding within the first day. The majority of cases reported the bleeding to be of mild severity. Our results showed that dental bleeding was greater among older age children. Also, dental bleeding was reported more frequently for "in-patients." This may be an artefactural finding as the parents were with the children in hospital and therefore this closer supervision may have led to a higher level of reports of bleeding.

Although it is considered not important, an inability to eat a normal meal was the major complaint reported in our study and higher than previously reported [3,6]. However, it was significantly reduced by the third day as reported by Holt and others [3]. This may be due to the high number of children complaining of sleepiness, drowsiness, sore throat, pain, and nausea/vomiting in the first day, which may affect their ability to eat. However, by the third day all complaints were reduced which enabled the children to eat normally. Dentists should assure parents and encourage the children to have a normal diet post-operatively.

About one third of the children complained of a sore throat in the first day. These results agrees with the findings of a study of GA service at one of London dental hospitals where sore throat was reported in 27% of the patients and was recorded as a late symptom (after home arrival) [3]. The reason for post-operative sore throat complaints may be due to the traumatic intubation by multiple attempt trials and double throat pack used by some of the pediatric dentists. Efforts should be directed to gentle manipulation of the throat tissue during intubation to avoid postoperative sore throat complaints.

Sleepiness was reported high in our study. Previously, it was reported that for every 10 min increase in anesthetic time the patient had a 15% increase of feeling sleepy [7]. The mean anesthetic time in our study was double the time that was previously reported [7], which might explain the higher percent of sleepiness reported in our study. Anesthesia duration as expected was positively influencing sleepiness and drowsiness.

Reports for post-operative nausea was rare, similar to previous studies [5,8]. However, vomiting was reported by 25% of the children in the first days, which is similar to Holt and others [3] but lower than Enger and Mourino [17], who operated for more than 3 hours and almost all of their patients were from ASA-PS higher than I. Our results found no association between vomiting complaint and children age, gender and medical status. This was against previous studies reports which correlate patient's age, gender and/or medical status with postoperative vomiting [4,18,19].

Fever was reported by 21% of the children in our study, which was found to be lower than previously reported (45%) [20]. Similar to present study, Morrow and others [20] found significant temperature elevation among young children. This could be explained by the longer duration of children pre-operative fasting regardless of their age and their inability to eat post-operatively. Those two reasons might lead to post-operative children dehydration and fever. Previous studies demonstrated the strong association between dehydration and fever [21,22]. Our results showed no associations between fever complaint neither with gender nor with medical status of the patients similar to Holan and others [23].

On the other hand, our study reported post-operative psychological changes in 24% of the children during the first day in the form of bad sleep and cry similar to Bridgman and colleagues findings [6], but less than behavioral changes reported in children following day case general surgery [24]. Since 96% of children in present sample received seveflurane, their behavioral changes were expected. Several studies found children anesthetized with sevoflurane exhibit more immediate postoperative distress than those anesthetized with halothane [25,26]. These suggestions of the relationship between psychological changes and anesthetic agents are supported by present results which showed a significant association between anesthetic duration and the reported complaints about psychological changes.

In associating postoperative morbidity with the nature of performed dental treatments, results showed that high number of dental procedures significantly increased postoperative dental pain. These findings could suggest the high expectation of postoperative dental pain among our children who received an extensive dental treatment.

As expected, and similar to previous reports [7,10,27], bleeding was reported more significantly among those who had a high number of extracted teeth (≥ 5 teeth).

Present results are in accordance to previous studies that the most common post-operative complaints were related to the performed dental treatment [3], but in contrast to those of Malhotra and others [19] who reported that post-operative complications were not significantly associated with the nature of performed dental treatment.

This is the first regional study to determine the type and frequency of postoperative complaints expected with pediatric dental GA procedure. Our sample from three governmental hospitals in Jeddah provided a preliminary local baseline data for pediatric dental GA procedure morbidity. The strength of our study came from its prospective design, which helped us in determining the post-operative experiences immediately and did not allow for poor recall of the events as in other retrospective studies. Future studies with broad inclusion of patients and hospitals could indicate the problems causation and its prevention. Present results are limited to the city of Jeddah, similar studies in other Saudi cities are recommended for the outcome to be representative of the population of Saudi children undergoing GA for dental procedures.

## Conclusion

In conclusion, Mild/moderate complaints were common in the first day post-operatively. But within the second post-operative day, most of the children regained their physical activity, and by the third day a significant reduction or disappearance of complaints were reported. The reported safety of the children operated under GA should encourage GA procedures use for recommended children.

## Competing interests

The authors declare that they have no competing interests.

## Authors' contributions

NF was the project director and edited the manuscript. RB carried out the study procedures and contributed in clarifying the manuscript. AB contributed in the study design and was the data manager. AA contributed in the study design and coordinated the study. All authors read and approved the final manuscript.

## Pre-publication history

The pre-publication history for this paper can be accessed here:



## References

[B1] Abushal M, Joseph OA (2000). The use of behavior management techniques by dentists in Saudi Arabia; A survey. Saudi Dental J.

[B2] GDC General Dental council (1997). Maintaining standards. London General Dental Council.

[B3] Holt RD, Chidiac RH, Rule DC (1991). Dental treatment for children under general anesthesia in day care facilities at a London dental hospital. Br Dent J.

[B4] Enever GR, Nunn JH, Sheehan JK (2000). A comparison of post-operative morbidity following outpatient dental care under general anaesthesia in paediatric patients with and without disabilities. Int J Pediatr Dent.

[B5] Vinckier F, Gizani S, Declerck D (2001). Comprehensive dental care for children with rampant caries under general anaesthesia. Int J Pediatr Dent.

[B6] Bridgman CM, Ashby D, Holloway PJ (1999). An investigation of the effects on children of tooth extraction under general anesthesia in general dental practice. Br Dent J.

[B7] Atan S, Ashely P, Gilthorpe MS, Scheer B, Mason C, Roberts G (2004). Morbidity following dental treatment of children under intubation general anesthesia in a day-stay unit. Int J Pediatr Dent.

[B8] Ersin NK, Oncag O, Cogulu D, Cicek S, Balcioglu ST, Cokmez B (2005). Postoperative Morbidities Following Dental Care under Day-Stay General Anesthesia in Intellectually Disabled Children. J Oral and Maxillofac Surg.

[B9] Patel RI, Hannallah RS (1998). Anesthetic complications following pediatric ambulatory surgery: a 3 year study. Anesthesiology.

[B10] Coulthard P, Rolfe I, Mackie C, Gazal G, Morton M, Jackson-Leech D (2006). Intraoperative local anaesthesia for paediatric postoperative oral surgery pain – a randomized controlled trial. Int J Oral Maxillofac Surg.

[B11] Haubek D, Fuglsang M, Poulsen S, Rolling I (2006). Dental treatment of children referred to general anesthesia association with country of origin and medical status. Int J Pediatr Dent.

[B12] North S, Davidson LE, Blinkhorn AS, Mackie IC (2007). The effects of a long wait for children's dental general anaesthesia. Int J Paediatr Dent.

[B13] O'Donnell A, Henderson M, Fearne J, O'Donnell D (2007). Management of postoperative pain in children following extractions of primary teeth under general anaesthesia: A comparison of paracetamol, Voltarol and no analgesia. Int J Pediatr Dent.

[B14] A Verbal Pain Scale. http://www.intelihealth.com/IH/ihtIH/WSI/29721/32087.html#verbal.

[B15] Ogg TW (1972). An assessment of postoperative out-patient cases. Br Med J.

[B16] Fung DE, Cooper DJ, Barnard KM, Smith PB (1993). Pain reported by children after dental extractions under general anesthesia: a pilot study. Int J Pediatr Dent.

[B17] Enger DJ, Mourino AP (1985). A survey of 200 pediatric dental general anesthesia cases. ASCD J Dent Child.

[B18] Moore JK, Moore EW, Elliott RA (2003). Propofol and halothane versus sevoflurane in pediatric day-case surgery Induction and recovery characteristics. Br J Anaesth.

[B19] Malhotra R, Rodd HD, Robertson S, North S, Davidson LE (2004). Factors affecting postoperative morbidity in children following dental general anesthesia. Int J Pediatr Dent.

[B20] Morrow JW, Seale NS, Berry CW, Love WD (1986). Incidence of temperature elevations after a full mouth dental rehabilitation under general anesthesia. ASDC J Dent Child.

[B21] Victoria M, Carvalho-Costa FA, Heimann MB, Leite JP, Miagostovich M (2007). Prevalence and molecular epidemiology of noroviruses in hospitalized children with acute gastroenteritis in Rio de Janeiro, Brazil, 2004. Pediatr Infect Dis J.

[B22] Fan JL, Cotter JD, Lucas RA, Thomas K, Wilson L, Ainlie PN (2008). Human cardiorespiratory and cardiovascular function during severe passive hyperthermia: effects of mild hypohydration. J Appl Physiol.

[B23] Holan G, Kadari A, Engelhard D, Chosack A (1993). Temperature elevation in children following dental treatment under general anesthesia with or without prophylactic antibiotics. Ped Dent.

[B24] Kotiniemi LH, Ryhänen PT, Moilanen IK (1997). Behavioural changes in children following day-case surgery: a 4-week follow-up of 551 children. Anaesthesia.

[B25] Breschan C, Platzer M, Jost R, Stettner H, Likar R (2007). Midazolam does not reduce emergence delirium after sevoflurane anesthesia in children. Paediatr Anaesth.

[B26] Keaney A, Diviney D, Harte S, Lyons B (2004). Postoperative behavioural changes following anesthesia with sevoflurane. Paediatr Anaesth.

[B27] Al-Bahlani S, Sheriff A, Crawford PJ (2001). Tooth extraction, bleeding and pain control. J R Coll Surg Edinb.

